# Superoxide Dismutase (SOD)-mimetic M40403 Is Protective in Cell and Fly Models of Paraquat Toxicity: IMPLICATIONS FOR PARKINSON DISEASE*

**DOI:** 10.1074/jbc.M115.708057

**Published:** 2016-03-07

**Authors:** Roberta Filograna, Vinay K. Godena, Alvaro Sanchez-Martinez, Emanuele Ferrari, Luigi Casella, Mariano Beltramini, Luigi Bubacco, Alexander J. Whitworth, Marco Bisaglia

**Affiliations:** From the ‡ Molecular Physiology and Biophysics Unit, Department of Biology, University of Padova, 35121 Padova, Italy,; the § MRC Centre for Developmental and Biomedical Genetics, Department of Biomedical Science, University of Sheffield, Sheffield S10 2TN, United Kingdom,; the ¶ MRC Mitochondrial Biology Unit, Cambridge Biomedical Campus, Cambridge CB22 LY, United Kingdom, and; the ‖ Department of Chemistry, University of Pavia, 27100 Pavia, Italy

**Keywords:** antioxidant, oxidative stress, Parkinson disease, superoxide dismutase (SOD), superoxide ion

## Abstract

Parkinson disease is a debilitating and incurable neurodegenerative disorder affecting ∼1–2% of people over 65 years of age. Oxidative damage is considered to play a central role in the progression of Parkinson disease and strong evidence links chronic exposure to the pesticide paraquat with the incidence of the disease, most probably through the generation of oxidative damage. In this work, we demonstrated in human SH-SY5Y neuroblastoma cells the beneficial role of superoxide dismutase (SOD) enzymes against paraquat-induced toxicity, as well as the therapeutic potential of the SOD-mimetic compound M40403. Having verified the beneficial effects of superoxide dismutation in cells, we then evaluated the effects using *Drosophila melanogaster* as an *in vivo* model. Besides protecting against the oxidative damage induced by paraquat treatment, our data demonstrated that in *Drosophila* M40403 was able to compensate for the loss of endogenous SOD enzymes, acting both at a cytosolic and mitochondrial level. Because previous clinical trials have indicated that the M40403 molecule is well tolerated in humans, this study may have important implication for the treatment of Parkinson disease.

## Introduction

Parkinson disease (PD)^3^ is an incurable chronic and progressive neurodegenerative disorder characterized by the preferential death of dopaminergic neurons in the midbrain area known as substantia nigra, resulting in a decrease of dopamine levels in its striatal projections. Although the discovery of monogenic, hereditable forms of the disease, which represent 5–10% of all cases, has been very important in helping to delineate the molecular pathways that lead to this pathology, PD is generally a sporadic neurological disorder. The etiology of idiopathic forms of PD is still poorly understood and aging is considered the most important risk factor. Strong evidence now exists to support a role for aberrant mitochondrial form and function, as well as increased oxidative stress in the progression of PD (1). For example, an increase in 8-hydroxy-2-deoxy guanosine, 4-hydroxy-2,3-nonenal, and protein carbonylation, which are respectively markers of DNA damage, lipid peroxidation, and protein oxidation, have all been detected in *postmortem* tissues from PD patients (2–5).

Oxidative damage occurs when the generation of reactive oxygen species (ROS) overcomes the elimination rate of the endogenous antioxidant system. The main cellular ROS are superoxide anion (O_2_^˙̄^), hydroxyl radical (OH^•^), and hydrogen peroxide (H_2_O_2_). Even though superoxide anion is relatively unreactive, it is considered the “primary” ROS because it can further interact with other molecules to produce more reactive “secondary” ROS, such as the hydroxyl radical (6). In cells, superoxide anions are mainly formed in mitochondria during oxidative ATP production, when a small leakage of electrons from the electron transport chain can directly react with oxygen to produce superoxide radicals (7). In addition to mitochondria, in dopaminergic neurons the auto-oxidation of dopamine contribute to cytosolic generation of superoxide and hydrogen peroxide (8).

Considering the potential toxicity related to physiological production of ROS, cells possess several endogenous antioxidant enzymes and low molecular weight reductants. Among the ROS-scavenging enzymes, superoxide dismutase enzymes (SODs) are often regarded as the first line of defense against ROS (9). These proteins convert naturally occurring superoxide radicals to molecular oxygen and hydrogen peroxide. Three different SOD isoenzymes, that are well compartmentalized, have been characterized in humans (see Zelko *et al.* (10) for a review). SOD1 is a copper/zinc protein located in the cytosol and in the mitochondrial intermembrane space, but is also present in peroxisomes and in the nucleus. SOD2 is a mitochondrial manganese enzyme, which is the main scavenger of superoxide anions produced during the mitochondrial oxidative phosphorylation. SOD3 is an extracellular copper/zinc protein, which, in contrast to intracellular SOD1 and SOD2, is expressed in only few cell types and tissues, such as vascular smooth muscular cells, lung, and plasma (10).

Pesticides represent one of the main factors involved in environmental chemical pollution (11). Epidemiological studies demonstrated that chronic exposure to pesticides, such as paraquat (PQ) and rotenone, is associated with a higher risk of developing PD (12–14). Consistently, two independent meta-analyses found an association between pesticides, in particular PQ, and the risk of PD (15, 16). PQ is able to enter dopaminergic neurons through a mechanism that involves the dopamine transporter DAT and the organic cations transporter-3 (17). Dopaminergic cell death induced by PQ is ascribed to the generation of ROS. Recently, it has been shown that PQ promotes oxidative damage both at the mitochondrial level and in the cytosol (18).

Despite the central role played by oxidative damage in the progression of PD, to date, the effects obtained with antioxidant therapies are modest (see Kanthasamy *et al.* (19) for a review). However, most studies do not target the primary cause of the oxidative stress, *i.e.* excessive superoxide anion production, but rather the downstream effects (production of hydrogen peroxide, hydroxyl radical or peroxynitrite). This is the case, for example, of α-tocopherol (vitamin E), ascorbic acid (vitamin C), creatine, and apocynin (19). A treatment strategy for oxidative stress is likely to be more effective if it targets the origin of ROS generation. As superoxide accumulation is the main mechanism involved in the subsequent formation of ROS, its catalytic elimination should have important cytoprotective effects.

In the present work we first assessed the potential protective role of SODs against PQ-induced toxicity in human SH-SY5Y neuroblastoma cell lines. We then investigated the therapeutic potential of the SOD-mimetic compound M40403, which has many properties that make it very attractive from a therapeutic point of view. Having verified the beneficial effects of superoxide dismutation in cells, we evaluated the effects using *Drosophila melanogaster* as *in vivo* model. Our data demonstrate that in *Drosophila* M40403 is able to compensate for loss of either Sod or Sod2, the homologous enzymes to human SOD1 and SOD2, acting both at a cytosolic and mitochondrial level, and to protect against oxidative damage induced by PQ treatment. In conclusion, in light of the association between PQ and PD, this work represents the first step in defining specific SOD-mimetic compounds as potential therapeutic agents to slow down PD progression.

## Experimental Procedures

### 

#### 

##### M40403 Synthesis

The synthesis of M40403 precursor *N*,*N*′-bis{(1*R*,2*R*)-[2-(amino)]cyclohexyl}-1,2-diaminoethanetetrahydrochloride was carried out as reported in the US patent 6214817 B1 without modifications. M40403 was then prepared following the conditions reported for the synthesis of either active and inactive complexes (20) the manganese complex was synthesized by binding of *N*,*N*′-bis{(1*R*,2*R*)-[2-(amino)]cyclohexyl}-1,2-diaminoethane and 2,6-pyridinedialdehyde to obtain the active compound.

##### Cell Culture

Human neuroblastoma SH-SY5Y cells (IST, Genova, Italy) were cultured in a mixture 1:1 of Ham's F12 and Dulbecco Modified Eagle Medium (Gibco®/Life Technologies) supplemented with 10% fetal bovine serum, in a 5% CO_2_ humidified incubator at 37 °C. The cell medium was replaced every 3 days, and the cells were sub-cultured once confluence was reached.

##### Cell Viability Assay

Cell viability was measured by colorimetric assay using the Cell Counting Kit-8 (CCK-8, Sigma) according to the manufacturer's instruction. Wild-type and transgenic cells were transferred on 96-well plates (10^4^ cell/well) in phenol red-free medium. One day after seeding, cells were treated for 24 h with 100–500 μm of PQ (Sigma-Aldrich); then they were incubated with 10 μl of CCK-8 solution for 4 or 6 h at 37 °C. The absorbance was measured at 460 nm using a plate reader (Victor^TM^ X3, Perkin Elmer).

##### FACS Analysis

Apoptosis was measured by Annexin V/Propidium Iodide (PI) double staining detection (BDPharmigen^TM^) using flow cytometry. Wild-type and stably transfected cells were cultured on 6-well plates. One day after seeding, cells were treated with 100–500 μm of PQ for 24 h. Then, cells were detached by 3 min treatment with papain protease (Worthington), centrifuged at 500 g for 5 min and resuspended in 500 μl of binding buffer (10 mm Hepes/NaOH, pH 7.4, 140 mm NaCl, 2.5 mm CaCl_2_). The cell suspension was then transferred into a 5 ml round-bottom tube and 1.5 μl of Annexin V-FITC (dilution 1:50) and 2 μl of PI (5 μg/ml) were added and incubated for 8 min at room temperature. Samples were analyzed by FACSCanto II flow cytometry (BD Bioscience) acquiring 10,000 ungated events. Annexin V^+^ PI^−^ cells represented the early apoptotic populations, Annexin V^+^ PI^+^ cells represented either late apoptotic or secondary necrotic populations while viable cells were Annexin V^−^ PI^−^.

##### roGFP Analysis

The expression vectors coding for cytosol and mitochondria-directed roGFP were obtained from Prof. James Remington's Lab, University of Oregon. SH-SY5Y cells (2 × 10^5^ cells) were transferred on poly-lysine pre-coated dishes (μ-Dish 35 mm, Ibidi) suitable for live imaging and transfected using Lipofectamine (Life Technologies) according to the manufacturer's instruction. After 24 h, cells were exposed to 500 μm PQ for 6 or 12 h in growing medium without phenol red. At the end of the treatment, images were collected with a Leica SP5 confocal microscope with 63× objective (oil immersion). Fluorescence was collected between 500–530 nm using 405 and 488 nm as excitation wavelength. To avoid photobleaching and/or laser-induced oxidation, images were acquired every 2 min using a wide pinhole and a fast scanning speed (256 × 256). Raw data were exported to ImageJ software. Each cells in the field was selected as region of interest (ROI) and the mean intensity of each ROI was then measured after appropriate background correction. Ratios between the values obtained by exciting at 405 and 488 nm, respectively, were normalized with respect to the ratios obtained in the presence of 1 mm H_2_O_2_ (100% oxidized state) and 4 mm DTT (0% oxidized state).

##### Mitochondrial Morphology

SH-SY5Y cells were transferred on poly-lysine-coated coverslips in 24 well plates (10^5^ cells per well) and transfected with an expression vector coding for a mitochondria-directed RFP, using Lipofectamine (Life Technologies) as transfection reagent according to manufacturer's instruction. After fixation, images were acquired using epifluorescence using a Leica 5000B with 100× oil objective. The data analysis was performed in a blind manner and reported as percentage of cells with tubular, intermediate, or fragmented morphology relative to total cell number.

##### Drosophila Strains and Culture Maintenance

Flies were raised on standard yeast-molasses-agar medium at 25 °C and 70% relative humidity in 12 h light/dark cycles. Only male flies were used in the experiments. A *white* (Dahomey) strain was utilized as wild-type control line (a gift from Linda Partridge, UCL). For all experiments employing GAL4 expression to drive UAS-transgenes, GAL4/+ were utilized as controls. The following strains were obtained from the Bloomington *Drosophila* Stock Center: UAS-Sod (33605), UAS-Sod2 (24494), da-GAL4 (5460), UAS-Sod-RNAi (24491), UAS-Sod2-RNAi (24489). TH-GAL4 was a gift from Serge Birman (21).

##### Western Blot Analysis

Proteins from untransfected and stably transfected cell extracts were subjected to SDS-PAGE (12%) and blotted onto PVDF membranes (Immobilion, Millipore). Blots were then incubated with rabbit polyclonal antibody for SOD1 or SOD2 (Prestige, Sigma) and mouse monoclonal antibody for β-tubulin (Sigma). The PVDF membranes were probed with horseradish peroxidase-conjugated anti-rabbit or anti-mouse IgG (Sigma). Immunoreactive proteins were visualized using enhanced chemiluminescence advance (ECL, GE Healthcare). Densitometry was carried out using Image J Software and the constitutively expressed β-tubulin protein was used as loading control.

##### Immunofluorescence

Untransfected and stably SOD1 or SOD2 transfected cells were plated on 15 mm glass coverslips pre-coated with poly-d-lysine. Twenty-four hours after seeding, cells were fixed with 4% paraformaldehyde, permeabilized with 0.1% Triton, and incubated with rabbit polyclonal antibody for SOD1 (Prestige, Sigma) or SOD2 (StressMarq, Bioscience Inc.) at a 1:200 dilution. To define the mitochondrial localization of overexpressed SOD2, samples were also incubated with mouse polyclonal succinate dehydrogenase subunit A (Santa Cruz) at a 1:200 dilution. Cells were subsequently incubated with secondary anti-rabbit or anti-mouse antibodies conjugated with Alexa Fluor-488 and Alexa Fluor-568 (Life Technologies) at a 1:200 dilution. Nuclei were counterstained using 0.16 μm Hoechst 33258 (Life Technologies) Coverslips were mounted with ProLong Gold Antifade (Life Technologies), and images were acquired using a Leica DM5000 epifluorescence microscope.

##### Quantitative Real-Time PCR

RNA was extracted using an RNeasy RNA purification kit (Qiagen), and cDNA was synthesized using a Protoscript II first-strand cDNA synthesis kit (New England BioLabs) according to the manufacturers' instructions from whole flies. Quantitative real-time PCR (qRT-PCR) was performed using a standard protocol using the 7900HT Fast Real-Time PCR System (Applied Biosystems) and Taqman probes, and each sample was normalized to the reference gene, *Rpl32*. Relative gene expression was calculated using the 2-ΔΔCT (cycle threshold) method.

The primers used for qRT-PCR are as follows: Rpl32: fw CACCGGAAACTCAATGGATACT, rev CACACAAGGTGTCCCACTAAT; probe: CCAAGAAGCTAGCCCAACCTGGTT; Sod: fw CCACTGTGCTGATCTACTCTATTT, rev CTAACAGACCACAGGCTATGTATT; probe: AGCACTACCCACTGGAGATATACAAACGA; Sod2: fw GCGAAATAACGAGAACGTAAGC, rev TTACGGGCCACGAACATATC; probe: TCGGGACTTAGCCTTATTAGCAGTCGA.

##### Survival Experiments

Groups of 20–25 one- to two-day-oldflies were starved for 4 h and then kept in vials with filter paper soaked with 5 mm PQ (plus 200 μm to 1 mm M40403 when required) in 5% sucrose. Surviving to the chemical treatment was determined every day for a 4 day-period. Experiments were repeated 4–6 times for control and experimental genotypes, and the mean and S.E. were calculated.

##### Locomotion Assay

Groups of 20–25 one- to two-day-old flies were starved for 4 h and then kept in vials with filter paper soaked with 1 mm PQ (plus 200 μm to 1 mm M40403 when required) in 5% sucrose. The filter paper was replaced every 2 days and the locomotion assays were performed after 5–7 days of treatment. The mobility of flies from each treatment group was assessed using a counter-current apparatus in a negative geotaxis climbing assay. Flies were placed in an empty plastic vial (2.5 cm diameter), gently tapped to the bottom, and the number of flies crossing a line at 8-cm height within a time period of 10 s was scored. Each animal was tested 5 times. The number of male flies tested per genotype was *n* > 150.

##### Statistical Analysis

Data were analyzed using GraphPad Prism 4 software. “*t* test,” one-way ANOVA followed by Bonferroni *post hoc* test or logrank test were used to determine whether groups were statistically different. *p* values < 0.05 were considered significant.

## Results

### 

#### 

##### PQ Treatment Increases Mitochondrial ROS Production

**FIGURE 1.**
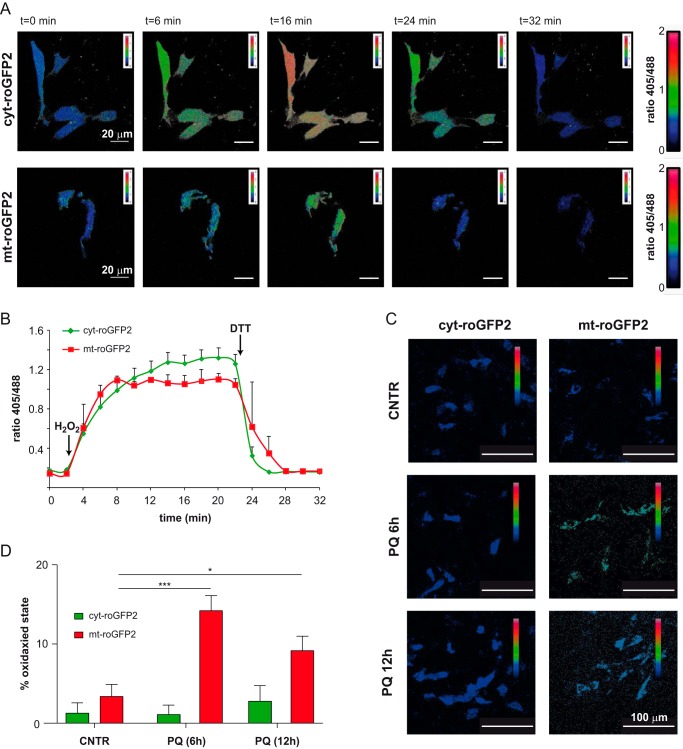
**PQ increases mitochondrial ROS production in SH-SY5Y cells.** *A*, SH-SY5Y cells expressing cyt-roGFP2 or mt-roGFP2 were excited at 405 and 488 nm, and the ratio of the emission in the green channel (500–530 nm) was calculated. Exemplifying images of the ratios obtained at representative time points are shown in pseudocolor calibrated by the color scale at the *far right. B*, time course of cellular redox changes monitored by changes in the ratio between fluorescence intensities obtained by exciting at 405 and 488 nm cyt-roGFP2 or mt-roGFP2-expressing cells. *C*, exemplifying pseudocolor ratio pictures of cells expressing cyt-roGFP2 or mt-roGFP2 recorded 6 and 12 h after PQ treatment (*D*). Fluorescence ratios (405/488) obtained for each sample were normalized with respect to the ratios corresponding to the 100% reduced and oxidized state. Data are reported as mean ± S.E. at least three independent experiments. Statistical significance was determined by *t* test comparing PQ-treated with PQ-untreated cells (*, *p* < 0.05; ***, *p* < 0.001).

To quantify alterations in the oxidative state induced by PQ and to define whether this process involves different cell compartments, we used a redox sensitive green fluorescent protein (roGFP2), which has two cysteine residues that form a disulfide bond under oxidizing conditions (22). This genetically encoded indicator allows the measurement of cellular redox state in real-time, regardless of the absolute levels of probe concentration, through ratiometric imaging of 405 nm *versus* 488 nm excitation (22). Two different variants of roGFP2 were used in the present work: one was cytosolic (cyt-roGFP2), while the other one was specifically targeted to mitochondria (mt-roGFP2) as previously reported (22). For each fluorescent probe, a calibration assay was first performed. The redox state was altered by the addition of 1 mm H_2_O_2_ and 4 mm DTT. As reported in Fig. 1, *A* and *B*, in the initial phase, unperturbed cells were in a predominantly reduced state. After the addition of 1 mm H_2_O_2_, the ratio (405/488) increased reaching a plateau within few minutes, in agreement with the peroxide-depending oxidation effect. Afterward, by adding 4 mm DTT the reduced state was restored shifting the fluorescence ratio to its minimum. After calibrating our system, this technique was used to assess the cellular redox state following PQ exposure. Considering that ROS production is, most likely, an early event that precedes cell damage, we treated cells with PQ for a relatively short period (6 and 12 h). While PQ was not able to increase the oxidative state in the cytosol, the treatment significantly increased mitochondrial ROS production after both 6 and 12 h (Fig. 1, *C* and *D*). Consistent with our results, in a recent study it has been showed that, after 24 h PQ exclusively increased mitochondrial ROS production, while only after 48 h a dose-response increase in both mitochondrial and cytosolic oxidative stress was observed in the surviving cells (18).

##### SOD2 but Not SOD1 Protects Against Paraquat-induced Toxicity

**FIGURE 2.**
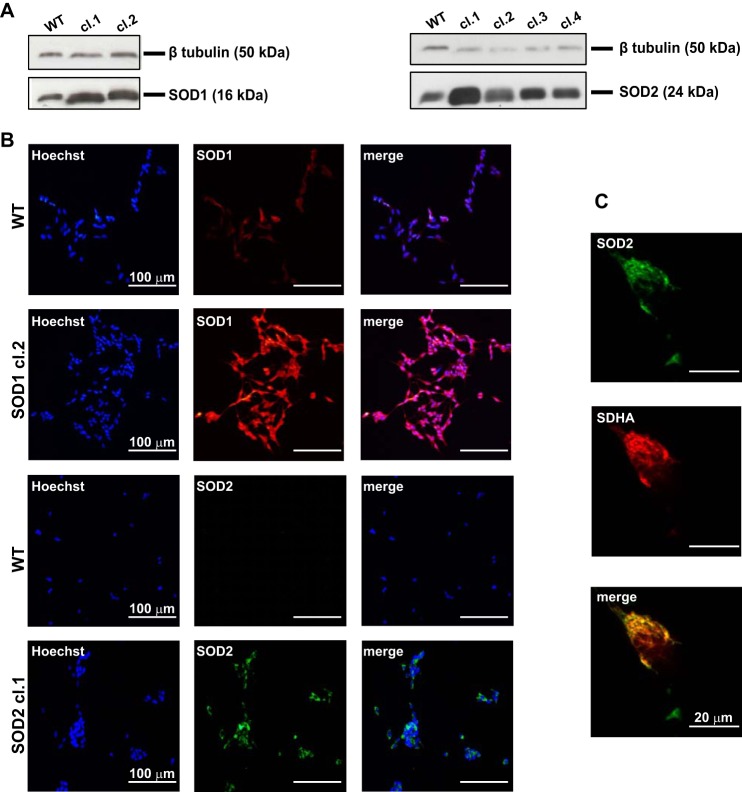
**SOD1 and SOD2 overexpression and localization in SH-SY5Y cell line.** *A*, Western blot analyses of SOD1 and SOD2 in stably transfected and untransfected SH-SY5Y cells. β-Tubulin signal was used as loading control. *B*, immunofluorescence microscopy. *Red staining* revealed that SOD1 was evenly distributed in stably transfected SH-SY5Y and confirmed overexpression compared with untransfected cells. Green staining confirmed the isolation of a unique clone and the increased expression of SOD2 protein in stably transfected SH-SY5Y compared with untransfected cells. *C*, SOD2 immunoreactivity (green fluorescence) showed excellent overlapping with Succinate dehydrogenase A (red fluorescence), which is located on the inner membrane of the mitochondria, indicating the mitochondrial localization of SOD2.

SH-SY5Y cell lines stably overexpressing human SOD1 or SOD2 were established and expression verified by Western blot analysis, Expression levels were quantified by densitometry and normalized with respect to tubulin (Fig. 2*A*). Clones with comparable SOD1 (clone 2) and SOD2 (clone 1) expression levels, 4.6 ± 1.3- and 5.7 ± 1.3-fold respectively, were selected for further analysis. Immunofluorescence analysis verified the cytoplasmic distribution of SOD1 and the mitochondrial localization of SOD2 (Fig. 2, *B* and *C*).

**FIGURE 3.**
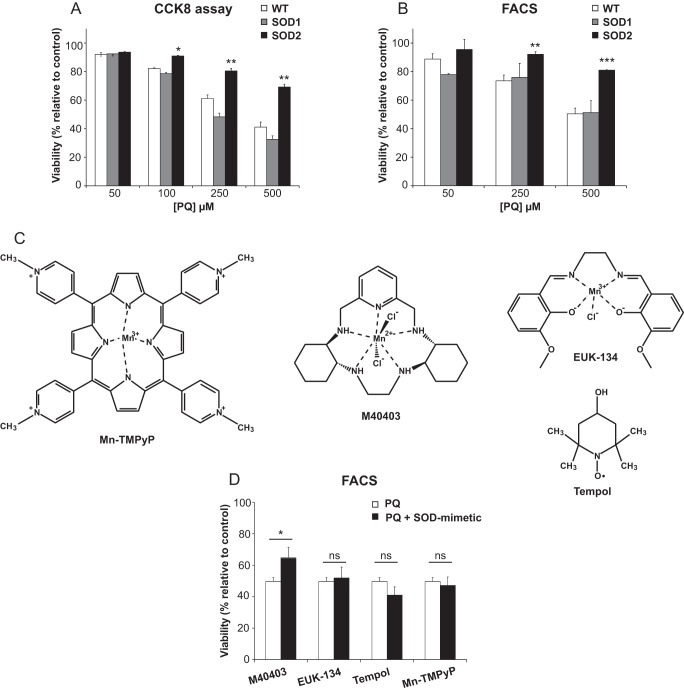
**SOD2 and M40403 protect SH-SY5Y cells against PQ toxicity.** Untransfected (WT), SOD1- and SOD2-overexpressing cells were treated with increasing amount of PQ. Histograms indicate the percentage of viable cells after treatment, relative to untreated cells used as control. Cell viability was measured by (*A*) the CCK8 colorimetric assay after 24 h of treatment or (*B*) through flow cytometry after 48 h of treatment. *C*, chemical structures of the SOD-mimetic compounds, belonging to four different classes of SOD-mimetics, that were used in the present work. *D*, SH-SY5Y cells were treated with 500 μm PQ in the presence of the indicated SOD-mimetic. Cell viability was measured through flow cytometry after 48 h of treatment. Histograms indicate the percentage of viable cells after treatment, relative to untreated cells. Data are expressed as mean of at least three different experiments ± S.E. Statistical significance was determined by *t* test comparing SOD-overexpressing cells with untransfected cells or SOD-mimetic-treated cells with untreated cells. (*, *p* < 0.05; **, *p* < 0.01; ***, *p* < 0.001.)

The potential protective role exerted by SODs against PQ toxicity was then evaluated. After incubation for 24 h with increasing concentration of PQ, SH-SY5Y cell viability, measured by a colorimetric (CCK8) assay, decreases in a dose-dependent manner (Fig. 3*A*). In agreement with previous results (23, 24), PQ treatment caused a dose-dependent cell death. With 500 μm PQ the measured viability was ∼40% of untreated cells. Interestingly, the overexpression of SOD1 and SOD2 produced markedly different effects. While SOD1 was unable to protect cells from the toxic insult induced by any concentration of PQ used, SOD2 provided significant protection at all PQ concentrations (Fig. 3*A*). An independent analysis based on fluorescence activated cell sorting (FACS) was performed to verify the results. As the events analyzed by FACS occurs in a time period subsequent to the metabolic dysfunctions observed by CCK8 assay, we prolonged the incubation for 48 h. As summarized in Fig. 3*B*, these results also showed a dose-dependent decrease in viability. Moreover, similar effects were also observed with SOD1 overexpression, while in the SOD2-overexpressing cells only at the highest amount of PQ used, some toxic effect became evident. In conclusion, these results indicate a selective effect exerted by SOD2 against PQ-induced toxicity.

##### Protective Role of the M40403 SOD-mimetic Compound

Considering the beneficial role observed for SOD2 in our cellular model, we analyzed the potential protective role of SOD-mimetic compounds. Four classes of molecules possessing SOD-like activity have been described until now (25), which include metalloporphyrin, nitroxides, Mn(III)-salen complexes and Mn(II)-pentaazamacrocyclic-based complexes. Using the cell model described above we studied compounds from each of the aforementioned classes, namely Mn(III)TMPyP, M40403, EUK-134, and Tempol (Fig. 3*C*). The effects of the SOD-mimetic compounds were analyzed via the FACS assay. As summarized in Fig. 3*D*, among the different drugs tested, only M40403 was able to significantly rescue PQ toxicity.

##### Mitochondrial Fragmentation Induced by PQ Is Rescued by SOD2 and M40403

**FIGURE 4.**
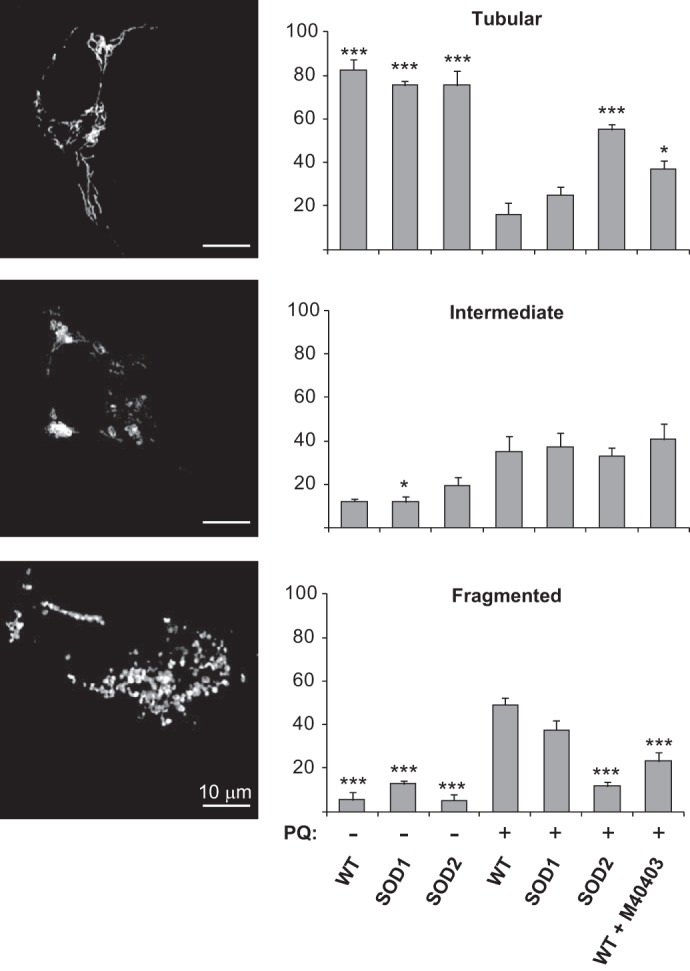
**SOD2 and M40403 rescue mitochondrial fragmentation induced by PQ.** Untransfected (WT), SOD1- and SOD2-overexpressing cells were transfected with mt-RFP to visualize the mitochondrial network. Cells were treated with 500 μm PQ for 24 h, in the presence or absence of the M40403 SOD-mimetic compound, and compared with untreated cells. Mitochondrial network was scored as tubular, intermediate, and fragmented. Tubular are elongated mitochondria with high connectivity, fragmented are mitochondria with a very small vesicular form and intermediate are a mixture of round and short mitochondria. The percentage of cells with a specific mitochondrial network was determined as a percentage of the total number of transfected cells counted. The data analysis was performed in a blind manner. Data are expressed as mean ± S.E. of at least three independent experiments. Statistical significance was assessed by one-way ANOVA with Bonferroni correction (*, *p* < 0.05; **, *p* < 0.01; ***, *p* < 0.001). For the sake of clarity, only statistical significance relative to PQ-treated WT cells is reported.

Mitochondria form a dynamic interconnected network that continuously undergo fission and fusion processes in order to maintain the proper morphology and functioning. Mitochondrial ROS production has been suggested to trigger mitochondrial fragmentation (26, 27). In light of the previous results, SH-SY5Y cells were transiently transfected to express a mitochondrial-targeted red fluorescent protein (mt-RFP), which allowed to monitor the mitochondrial morphological changes through fluorescence microscopy. As reported in a previous work (28), we classified mitochondria as tubular, intermediate and fragmented. Tubular are elongated mitochondria with high connectivity, fragmented are very small and round mitochondria and intermediate are a mixture of circular and shorter tubular mitochondria. As shown in Fig. 4, ∼80% of SH-SY5Y wild-type cells displayed mitochondria with a tubular shape, while only a small fraction has mitochondria with an intermediate or fragmented morphology. A similar morphological feature was observed in SOD1 and SOD2-overexpressing cells indicating that the overexpression of these enzymes does not perturb the mitochondrial network. When wild-type cells were treated with PQ we observed a strong shift toward fragmentation. We also observed an increase of the intermediate morphology in comparison to our controls. While the overexpression of SOD1 did not significantly affect the mitochondrial network in cells treated with PQ, the overexpression of SOD2 strongly reduced fragmentation, restoring a more tubular network. Interestingly, the addition of M40403 to the growth medium also protected mitochondria from the morphological changes induced by PQ.

##### Sod-mediated Protection Against PQ Toxicity in Drosophila melanogaster

**FIGURE 5.**
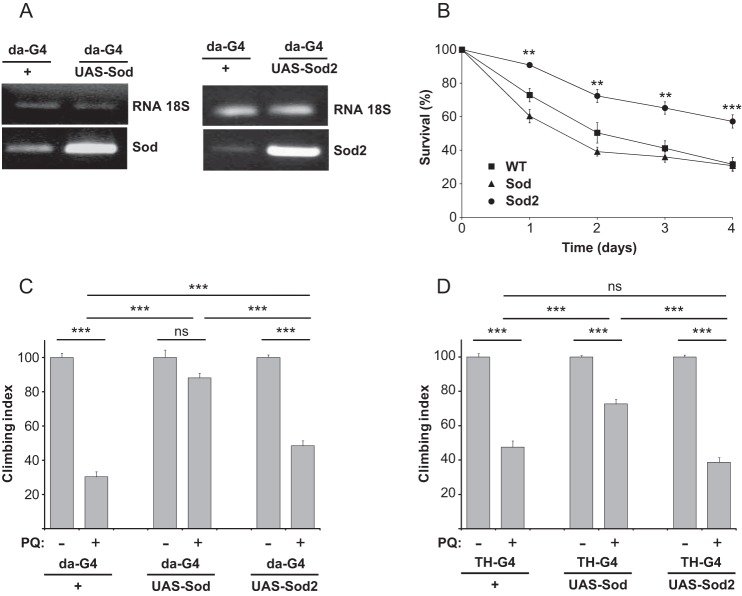
**Sod and Sod2 differentially protect *Drosophila* against PQ toxicity.** *A*, ubiquitous Sod and Sod2 overexpression in flies. Semi-quantitative PCR analysis of Sod and Sod2 levels. The da-GAL4/UAS system was used to ubiquitously drive the expression of either Sod or Sod2. 18S rRNA signal was used as loading control. *B*, survival of da-GAL4/+ (WT), da-GAL4>Sod and da-GAL4>Sod2 flies was monitored upon exposure to 5 mm PQ. *C* and *D*, locomotion behavior of flies was measured after exposure to a sublethal concentration (1 mm) of PQ for 7 days. Data are expressed as mean ± S.E. Statistical significance was determined by one-way ANOVA with Bonferroni correction (*, *p* < 0.05; **, *p* < 0.01; ***, *p* < 0.001).

The effects of the overexpression of SOD enzymes were also evaluated *in vivo*, using *Drosophila melanogaster*. The use of *Drosophila* presents several advantages. The rapid reproductive cycle and the short lifespan of flies readily allow the analysis of age-related events on a large number of individuals. Moreover, particularly relevant for this study, it is possible to target the expression of transgenes into specific cell types, such as dopaminergic neurons. Several studies have already analyzed the effect of PQ toxicity on fly survival (29, 30), including in models of PD (31). *Drosophila* express homologs of both SOD1 and SOD2, called Sod and Sod2 respectively. We used the GAL4/UAS system to drive the overexpression of either Sod or Sod2 in a tissue-specific manner (32). First, the proteins were expressed ubiquitously via the da-GAL4 driver. The overexpression was evaluated by semi-quantitative PCR analysis on fly lysate extracts and quantified through densitometry and normalized with respect to 18S rRNA (Fig. 5*A*). The overexpression levels of Sod and Sod2 in comparison to wild-type flies were 9 ± 2 and 9 ± 4, respectively. The acute treatment of 5 mm PQ causes ∼30% of da-GAL4/+ flies died after 1 day and more than 70% after 4 days (Fig. 5*B*). The overexpression of Sod did not provide any significant protection, in agreement with a previous work (33). Consistent with the results obtained in our cellular model, the ubiquitous overexpression of Sod2 made the flies significantly more resistant to PQ toxicity: more than 60% of flies were still alive after 4 days of treatment. These results suggest that the damage induced by 5 mm PQ is likely to compromise the organismal viability much more extensively at the mitochondrial level than the cytosol since only Sod2 overexpression resulted in rescue.

To analyze more in depth the neurotoxic effects associated to a chronic exposure of PQ, we treated flies with 1 mm PQ for 7 days, a sub-lethal condition. Defects in motor behavior were then analyzed using a negative-geotaxis climbing assay. After PQ treatment, control flies showed a strong impairment in locomotion (Fig. 5*C*). Interestingly, the ubiquitous overexpression of Sod via the da-GAL4 driver was able to almost completely rescue this motor dysfunction. In contrast, even though Sod2 overexpression improved the behavioral phenotype in a statistically significant manner, the rescue was only partial and much lower than with Sod. These results suggest that following chronic exposure to PQ, cytosolic production of superoxide lead to behavioral defects in flies. As chronic exposure to PQ has been associated to PD, we then analyzed the effects of the selective over-expression of Sods in the dopaminergic neurons using the TH-GAL4 driver. Once again, after the exposure to PQ the locomotion behavior of control flies was significantly reduced (Fig. 5*D*). Overexpressing Sod2 did not improve the motor dysfunction while an increased amount of Sod was able to significantly counteract the oxidative damage specifically induced by PQ in the dopaminergic neurons. In conclusion, in the presence of 1 mm PQ the damage appears to be mostly related to the cytosolic production of superoxide radical, which interferes with the correct functioning of neurons.

##### Protective Role of the M40403 SOD-mimetic Compound in Drosophila

**FIGURE 6.**
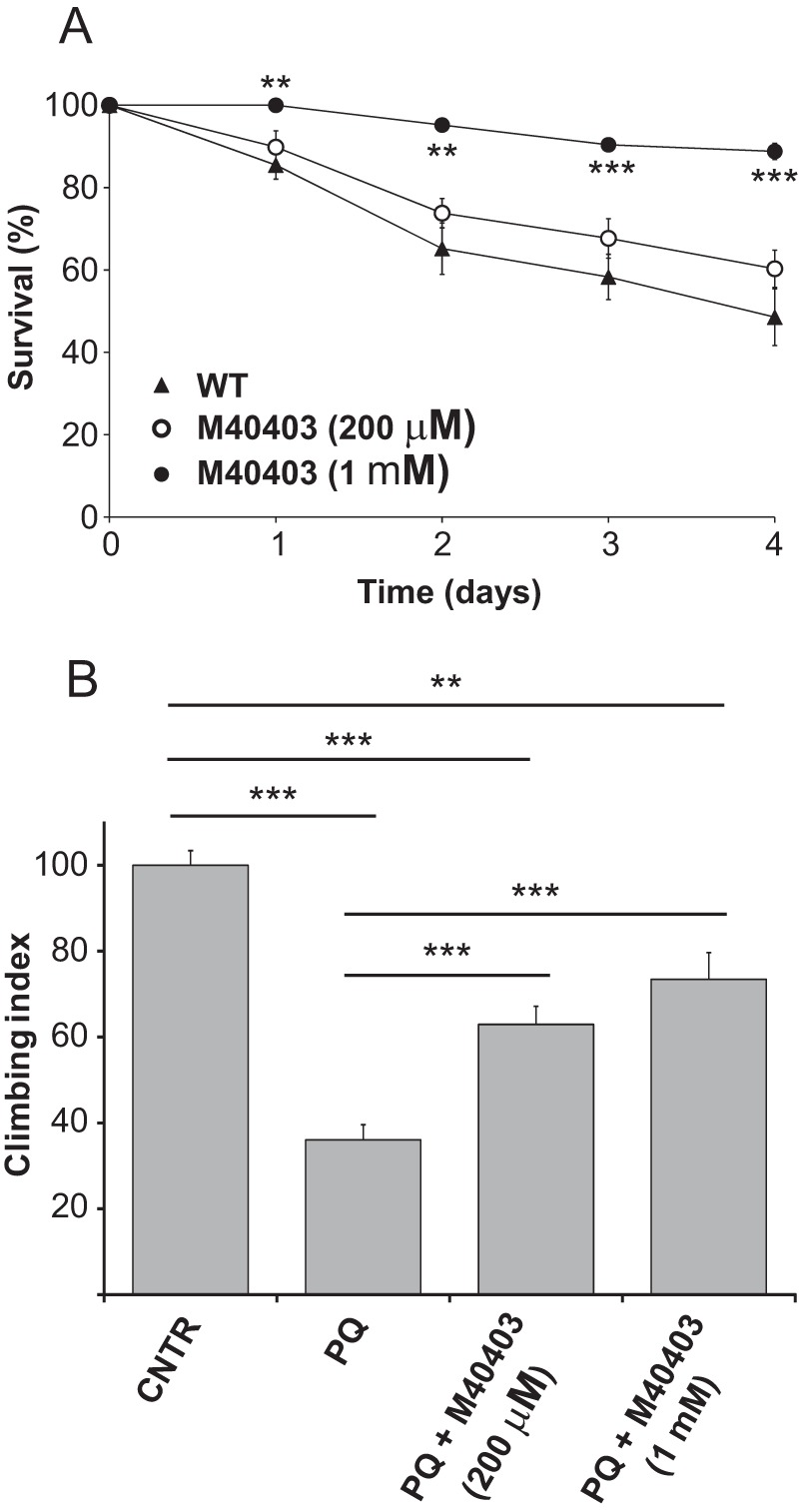
**M40403 protects *Drosophila* against PQ toxicity.** *A*, survival of wild type *Drosophila* (*w,* Dah) was monitored upon exposure to 5 mm PQ, in the absence or presence of different amounts of M40403. *B*, locomotion behavior of wild type flies was analyzed after treatment with a sublethal concentration (1 mm) of PQ for 5 days, in the absence or presence of different amounts of M40403. Data are expressed as mean ± S.E. Statistical significance was determined by one-way ANOVA with Bonferroni correction (*, *p* < 0.05; **, *p* < 0.01; ***, *p* < 0.001).

The protection of M40403 was also tested *in vivo* in wild-type flies. First, fly survival was evaluated by exposing flies to 5 mm PQ in the absence or presence of M40403. As before, survival was strongly affected by the presence of 5 mm PQ. However, the presence of M40403 increased fly survival in a dose-dependent manner and at the highest concentration used (1 mm) the rescue was almost complete (Fig. 6*A*). The locomotion behavior of flies treated with 1 mm PQ for 7 days was then analyzed in the absence or presence of M40403. Again, the climbing ability of flies was strongly affected by the exposure to PQ (Fig. 6*B*), but the presence of M40403, at both concentrations tested, resulted in a significant improvement of the climbing ability.

##### Lethality Rescue by M40403 in Sod- and Sod2-knockdown Flies

**FIGURE 7.**
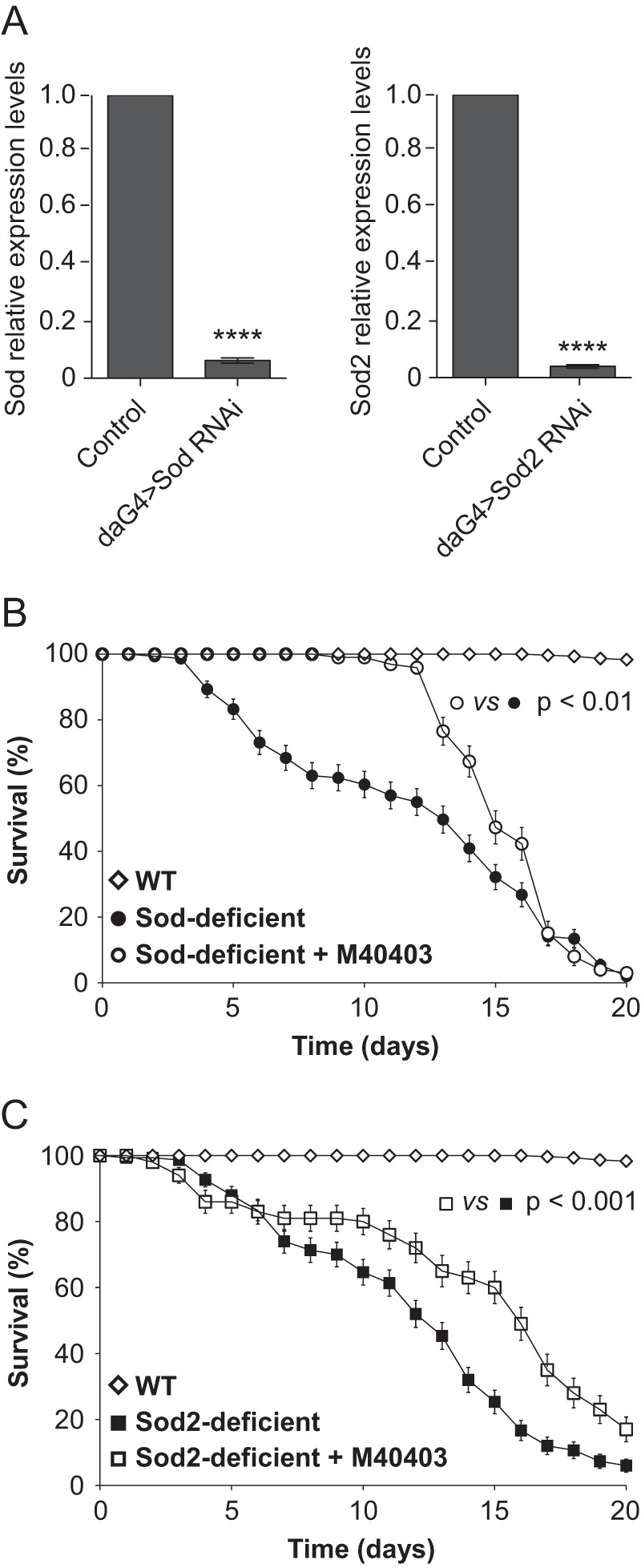
**The M40403 SOD-mimetic rescues the lethality in Sod- and Sod2-deficient flies.** *A*, quantitative RT-PCR analysis of endogenous Sod and Sod2 transcript levels. Sod- and Sod2-knockdown flies were generated through the expression of a UAS inverted repeat transgene, to induce RNA interference, via the ubiquitous da-GAL4 driver. Significance was determined by Student's *t* test (****, *p* < 0.0001). The survival of (*B*) Sod- or (*C*) Sod2-deficient flies was measured over a period of 20 days in the absence or presence of 1 mm M40403. The survival distribution of the untreated and M40403-treated flies was compared using the logrank test.

While from the results obtained in the presence of the higher concentration of PQ used it appears evident that M40403 is able to act similarly to SOD2, to infer its cytosolic activity from our data is more difficult. To address this important question, we evaluated whether the drug could affect fly survival upon Sod- and Sod2-knockdown, mediated via transgenic RNAi lines coupled with the da-GAL4 driver. The gene silencing was evaluated by qRT-PCR. As Shown in Fig. 7*A* the down-regulation of Sod and Sod2 transcripts was remarkably effective (90–95%). Moreover, the level of these transcripts was attenuated to a equivalent extent. The ubiquitous down-regulation of the enzymes has been described to lead to early adult mortality and elevated endogenous oxidative damage production (34, 35). In our experimental conditions, the viability of control flies is basically not affected during the experimental time-course. On the contrary, the survival of flies rendered deficient for either Sod or Sod2 is comparably much shorter than our control, with a median survival of 13 days. The co-treatment of flies with M40403 increased the survival for both genotypes, but with differential effects. In the case of Sod-deficient flies, the presence of M40403 appeared to be strongly protective in the first period of treatment (12 days), after which the survival rapidly decreased reaching the values similar to the untreated flies (Fig. 7*B*). In contrast, the drug was not able to reduce the mortality of Sod2-deficient flies in the first period of treatment but showed protection starting from the 6^th^ day until the end of the experiment (Fig. 7*C*). The survival distribution of the untreated and M40403-treated flies was compared through the logrank test, which indicated that in both cases the two curves are significantly different (*p* = 0.004 and *p* < 0.001 for Sod-and Sod2-knockdown flies, respectively). In conclusion the data presented here indicate that the SOD-mimetic molecule M40403 is able to partially rescue, *in vivo*, the loss of either Sod or Sod2, and suggest that it can act both at cytosolic and mitochondrial level.

## Discussion

Increased production of ROS and/or decreased capacity of antioxidant defense can disrupt oxidative balance and damage many components of the cell, including lipids, proteins, and DNA. It is well established that oxidative damage is closely linked to the progression of PD (1). Exposure to pesticides, such as PQ and rotenone, has been associated with a higher risk of developing PD (36), and increasing evidence exists on the induction of oxidative damage by PQ. Accordingly, PQ is widely used to generate neurotoxin-based models of sporadic PD (37–39).

In this study, we investigated the beneficial effects of SOD enzymes and SOD-mimetic compounds. The picture that emerges from all the experiments presented here emphasizes the role of both cytosolic and mitochondrial SODs in protecting cells against superoxide overproduction. Specifically, our *in vitro* analyses demonstrated that PQ promotes oxidative stress at mitochondrial level, which, in turn, impacts on the morphology of these organelles and, ultimately, on cell viability. These observations explain the selective protection exerted by SOD2, which resides directly on the site of ROS production. A similar picture emerges in flies treated with high concentrations of PQ (acute treatment), highlighting once again the important role played by SOD2 in protecting against oxidative damage. On the contrary, the situation observed in flies at sub-lethal concentrations of PQ indicates that only the overexpression of SOD1 is able to rescue the PQ-associated toxicity, while SOD2 appears ineffective. Interestingly, this is also true when SOD1 is specifically expressed in dopaminergic neurons, the neuronal population mainly affected in PD. In this condition (chronic treatment), endogenous SOD1 and SOD2 are probably able to counteract both cytosolic and mitochondrial production of superoxide in most tissues. Nevertheless, consistent with the notion that dopaminergic neurons are particularly vulnerable to oxidative damage conditions (40), other cytosolic processes inside these neurons, such as dopamine oxidation, may amplify the toxicity derived from an elevated production of free radical species. In support of this, a previous study showed that when PQ was administered with a non-toxic concentration of DA, significant increases in ROS levels and cell death were detected, suggesting that DA itself may contribute to the vulnerability of DA neurons to PQ toxicity (17). Moreover, consistent with this interpretation, the overexpression of the vesicular monoamine transporter, which reduces the cytosolic level of dopamine by sequestering it inside synaptic vesicles, has been described to protect from neurotoxin-induced degeneration of dopaminergic neurons, both in flies and mice (41, 42).

Wherever the origin of ROS production is, at mitochondrial level or in the cytosol, a very considerable result presented in this work, is the ability of the SOD-mimetic compound M40403 to prevent the PQ-induced toxicity. Not only M40403 showed significant beneficial properties in our cellular model, but, when tested *in vivo*, it succeeded in rescuing the lethality induced by elevated concentration of PQ. Moreover, in the presence of a sub-lethal concentration of PQ, M40403 was also able to improve the locomotion behavior of flies. The protective effects of M40403, both in the presence of acute and chronic PQ treatments, derive, most probably, from its ability to act *in vivo* both at cytosolic and mitochondrial level, as we demonstrated by using either Sod or Sod2 knockdown flies. This behavior is of particular importance in the context of PD, where cytosolic processes can contribute to and exacerbate the production of superoxide radicals and where antioxidants targeted to mitochondria failed to show any benefit in clinical trials (43)

In the rational design of potential therapeutic molecules to combat PD, at least two additional aspects must be considered. The first is their ability to cross the brain blood barrier; the second is the absence of any toxicity. The *in vivo* distribution of M40403 has been described in rats, where the drug, 6 h after injection, was found widely distributed (44). Its presence in the brain clearly indicated its ability to cross the brain blood barrier. By monitoring the intact Mn(II)-complex in biological samples, the long lasting stability of M40403 was also demonstrated (44). Furthermore, results of phase I and phase II clinical trials that have already been performed in ∼700 subjects/patients using an intravenous formulation of M40403, indicate that it is safe and well-tolerated (45).

In conclusion, the M40403 molecule has unique properties that make it very attractive from a therapeutic point of view. M40403 does not just act as a free radical scavenger, but, like the native enzymes, is able to catalytically dismutate superoxide. As demonstrated in this work, it is able to rescue the toxicity induced by the attenuation of either SOD1 or SOD2, indicating that it can act both a cytosolic and mitochondrial level. The molecule is water-soluble so that it can be orally administered, but at the same time it is able to cross the blood brain barrier and to accumulate in the brain (44). Even though the following aspect was not considered in this work, M40403 has been originally designed with the aim to counteract inflammatory processes (see Salvemini *et al.* 46 for a review) rather than oxidative stress, and until now its clinical application is restricted to anti-inflammatory therapy (45). Considering that microglia activation is believed to contribute to the progression of PD, it is highly probable that the beneficial effects of M40403, described here, are underestimated.

In light of these considerations and of the data presented in this work, the possibility to explore a novel use of SOD-mimetic compounds as a disease modifying treatment for PD becomes very attractive. More specifically, SOD-mimetic compounds belonging to the M40403 family could be evaluated for their use as a complementary therapy against PD, in addition to the currently adopted treatments.

## Author Contributions

R. F. performed most of the cellular experiments. V. K. G. and A. S. M. performed most of the experiments in *Drosophila*. E. F. and L. C. designed and performed the synthesis of M40403. M. Be. and L. B. contributed to the conception of the study and to the revision of the paper. A. J. W. and M. B. designed and coordinated the study and wrote the paper.

## Footnotes

3 The abbreviations used are:

PD: Parkinson disease
CCK-8: cell counting kit-8
FACS: fluorescence activated cell sorting
PI: propidium iodide
PQ: paraquat
qRT-PCR: quantitative real-time PCR
ro-GFP: redox-sensitive green fluorescent protein
ROS: reactive oxygen species
SOD: superoxide dismutase.
